# Thermoelectric and electronic transport properties of thermal and plasma-enhanced ALD grown titanium nitride thin films

**DOI:** 10.1039/d5na00914f

**Published:** 2025-12-04

**Authors:** Priyanka Goel, Christoffer Kauppinen, Ramesh Raju, Ilkka Tittonen

**Affiliations:** a Department of Electronics and Nanoengineering, Aalto University Espoo 00076 AALTO Finland Priyanka.goel@aalto.fi; b VTT Technical Research Centre of Finland Ltd Tietotie 3 Espoo FI-02044 VTT Finland

## Abstract

Titanium nitride (TiN) thin films demonstrate high electrical conductivity and thermal stability up to 400 °C in ambient conditions, with stability extending to 600–800 °C under inert or vacuum environments. Unlike many metals and transition metal nitrides, TiN combines high carrier mobility with moderate carrier concentration, making it ideal for thermal management and power-efficient applications in nanoelectronics and energy harvesting. This study systematically investigates the thermoelectric and electronic transport properties of TiN films grown by plasma-enhanced atomic layer deposition (PEALD), comparing them to those produced using traditional thermal atomic layer deposition (thermal ALD). These properties are studied as a function of growth temperature and the number of growth cycles. In particular, TiN films deposited by PEALD at 400 °C for 2000 ALD cycles exhibited a remarkable power factor of 512 µW m^−1^ K^−2^ at room temperature compared to a power factor of 4.95 µW m^−1^ K^−2^ measured for thermal ALD films fabricated under the same deposition conditions. Additionally, thermal conductivity was also measured for thicker TiN films (86 nm), yielding values of 26.96 W m^−1^ K^−1^ for PEALD and 7.01 W m^−1^ K^−1^ for thermal ALD, marking the first such report for ALD-grown TiN. These values offer an upper estimate of the thermal behavior in thinner films. Based on these measured properties, the thermoelectric figure of merit (*zT*) at room temperature was calculated to be 0.0056 for PEALD TiN films which is significantly higher than the value of 0.0002 obtained for thermal ALD TiN films. Our findings provide critical insights into transport properties of TiN, offering guidance for the development of conductive nanolayers in thermoelectric, nanoelectronic, and on-chip cooling applications, where precise control over thermal and electronic behavior is vital, thereby expanding the relevance of ALD TiN in high-performance applications.

## Abstract 2

PEALD-TiN process enhances thermoelectric power factor and reduces resistivity *via* optimized growth temperature and number of cycles.

## Graphical abstract

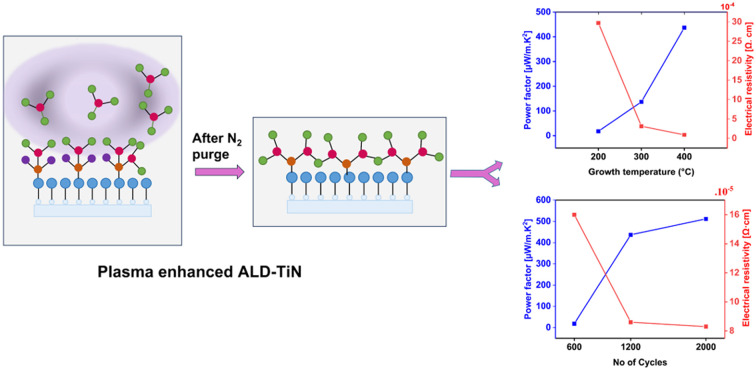

## 1 Introduction

Titanium nitride (TiN), a member of the transition metal nitrides, has attracted significant attention due to its many impressive physical properties and diverse applications^1,2^ across energy,^3–5^ plasmonics,^2,6^ optoelectronics,^7–9^ semiconductors,^10^ energy storage^11–13^ and electronics technologies.^14,15^ Specifically, it is known for its exceptional mechanical hardness (20 GPa)^16,17^ and corrosion resistance.^18,19^ These properties have enabled its widespread use as a protective coating in cutting tools^20–22^ and high-stress environments,^23,24^ where durability and stability are paramount. Furthermore, TiN has a high melting temperature, exceeding 2900 °C, which makes it ideal for high-temperature applications.^25–27^ Unlike traditional III-nitride semiconductors such as GaN that prefer to exhibit the wurtzite crystal structure, TiN crystallizes in a rock salt structure. Its partially filled Ti 3d conduction band, combined with a high density of intrinsic defects that degenerately dope the material, leads to metal-like electrical conductivity. Together with its strong covalent–metallic bonding, this structure also provides excellent mechanical strength even at elevated temperatures. Due to the impressive conductivity of TiN,^28^ it is widely adopted in semiconductor microelectronics as a metal gate barrier,^29^ where it is often deposited with atomic layer deposition (ALD). Furthermore, TiN's stability at high temperatures has led to its investigation in photothermal catalysis^30,31^ and solar-driven processes.^32^

Despite its well-established use in various fields, the potential of TiN in thermoelectric applications has remained largely unexplored. In contrast, other transition metal nitrides, such as scandium nitride (ScN)^33–35^ and chromium nitride (CrN),^36–39^ have gained considerable interest to their thermoelectric properties. However, some analyses have reported that the thermal conductivity of TiN is moderate^40^ rather than exceptionally high, but key parameters such as the Seebeck coefficient, electrical conductivity, and other electronic transport properties have been underexamined, highlighting a significant gap in understanding its potential applications. This knowledge gap opens a valuable opportunity to explore TiN for thermoelectric energy conversion, particularly for waste heat recovery applications.

This study investigated of the thermoelectric and electronic properties of TiN thin films deposited *via* atomic layer deposition (ALD), a technique that enables precise control over film thickness with excellent uniformity. We evaluated thermoelectric performance of TiN films including the Seebeck coefficient, electrical conductivity, thermoelectric power factor, thermal conductivity, along with Hall mobilities, and carrier concentration under varying deposition conditions.

## 2 Results and discussion

### 2.1 Thin film growth and structural properties

**Fig. 1:**
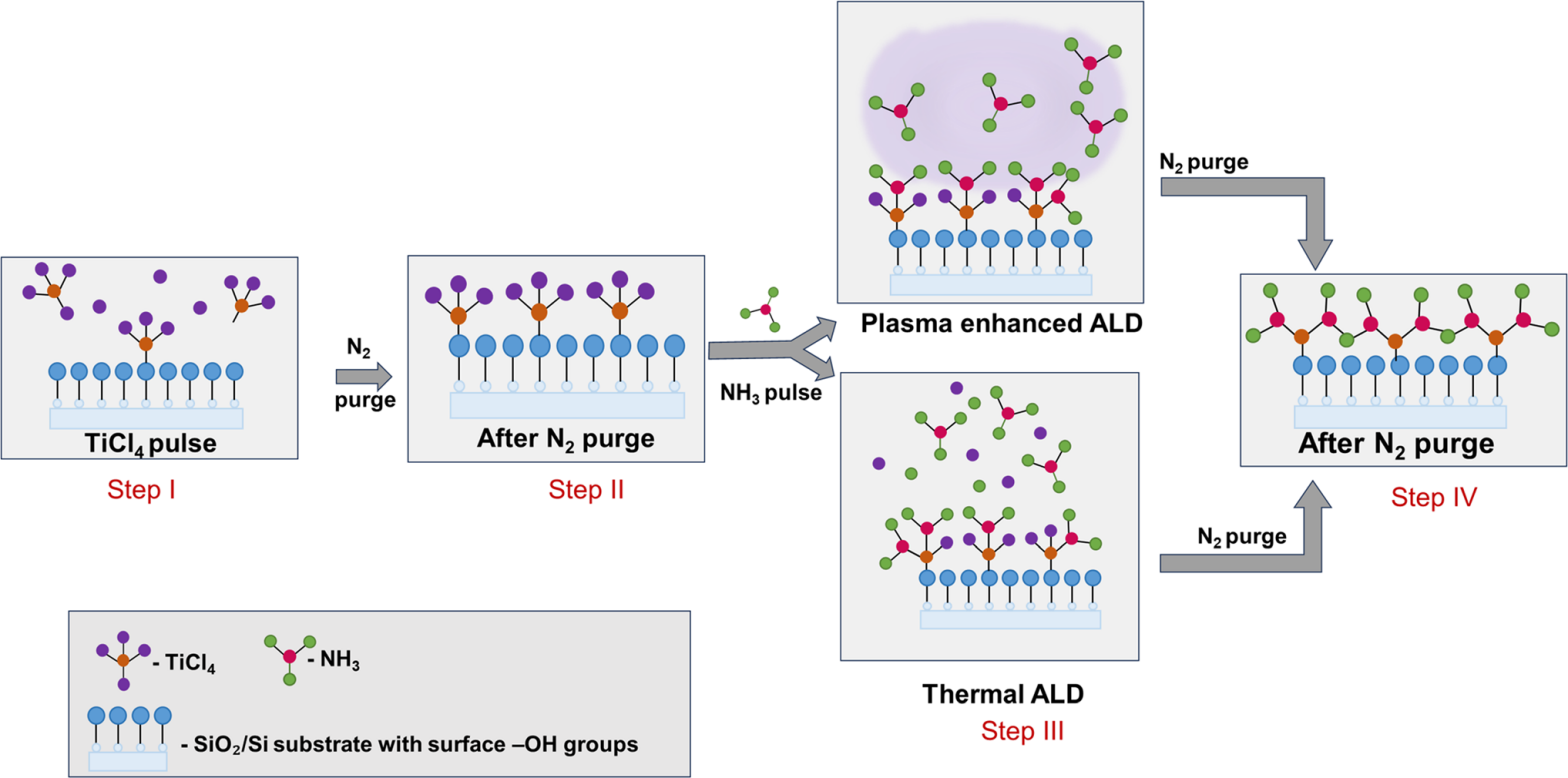
Illustration showing growth of TiN thin film using PEALD and thermal ALD technique.

TiN thin films were deposited at different temperatures with varying thickness using both the PEALD and thermal ALD techniques. The growth process is depicted in Fig. 1. As illustrated in the scheme, the process involved alternating exposures of the substrate to TiCl_4_ and NH_3_, separated by inert N_2_ purges. The process begins with the surface functionalized with reactive groups, such as hydroxyls (–OH), which are depicted as blue spheres in Fig. 1. These –OH groups act as reactive sites for the adsorption of TiCl_4_ molecules onto the surface, where only a monolayer adheres due to the self-limiting nature of ALD. When TiCl_4_ is introduced into the reaction chamber, it selectively chemisorbs onto these active sites, forming Ti–O surface bonds as shown in step I of Fig. 1, while releasing HCl as a by-product. The unreacted TiCl_4_ is then removed by purging with N_2_ (as shown in step II of Fig. 1). The substrate is then exposed to gaseous or plasma activated NH_3_, which reacts with chemisorbed TiCl_4_ to form TiN (step III – Fig. 1), with the HCl (reaction product) purged away. In thermal ALD, the reaction relies solely on thermal energy to activate the surface chemistry, resulting in layer-by-layer growth of TiN while in PEALD, NH_3_ is introduced in a plasma-activated state, generating reactive species (*e.g.*, NH_2_, H radicals) that enhance surface reactivity. The final surface is again purged with N_2_ to remove any leftover HCl and unreacted species. This cyclic and self-limiting process^41^ allows precise atomic level control over film thickness.

The thickness of TiN thin films deposited *via* PEALD and thermal ALD was measured using ellipsometry for 1200 ALD cycles at various deposition temperatures, with the results summarized in Table 1. At a deposition temperature of 400 °C, PEALD produced a film with a thickness of 31 nm, while thermal ALD produced a significantly thinner film of 12 nm under identical conditions. These values correspond to a growth per cycle (GPC) of 0.26 Å per cycle for PEALD and 0.10 Å per cycle for thermal ALD, showing a considerably higher deposition rate for the plasma-enhanced process. The enhanced deposition rate in PEALD can be attributed to the creation of reactive nitrogen species in the plasma, which facilitates more efficient surface reactions and incorporation of nitrogen into the TiN film in contrast to thermal ALD, where the ammonia reacts only *via* thermal reactions. To validate the consistency of the growth rate, additional film thickness measurements were conducted across different numbers of ALD cycles, as summarized in Table 2. The results indicated that at 400 °C the GPC for PEALD was approximately three times higher than that of thermal ALD. Interestingly, the PEALD process exhibited a slightly higher GPC at lower cycle numbers (600 cycles), pointing to surface-enhanced growth mechanism, as reported in previous studies.^41^ In contrast, thermal ALD appeared to have the opposite behavior with higher GPC in higher cycle numbers indicating surface-inhibited growth.^41^ It was interesting that for the same ALD material using the same gases the substrate surface interacted in opposite ways for thermal process compared to a plasma-enhanced process.

**Table 1:** Thickness and GPC of PEALD and thermal ALD at different temperatures for 1200 ALD cycles

Temperature	PEALD		Thermal ALD	
	Thickness	GPC	Thickness	GPC
200 °C	20 nm	0.17 Å	No deposition	0 Å
300 °C	31 nm	0.26 Å	1.2 nm	0.01 Å
400 °C	31 nm	0.26 Å	12 nm	0.10 Å

**Table 2:** Thickness and GPC of PEALD and thermal ALD at 400 °C for different no. of ALD cycles. The GPC values reveal distinct growth behaviors: PEALD showed substrate-enhanced growth, while thermal ALD exhibits substrate-inhibited growth

No. of cycles	PEALD		Thermal ALD	
	Thickness	GPC	Thickness	GPC
600	17.7 nm	0.30 Å	5.8 nm	0.10 Å
1200	31.0 nm	0.26 Å	12.0 nm	0.10 Å
2000	50.4 nm	0.27 Å	24.1 nm	0.12 Å

**Fig. 2:**
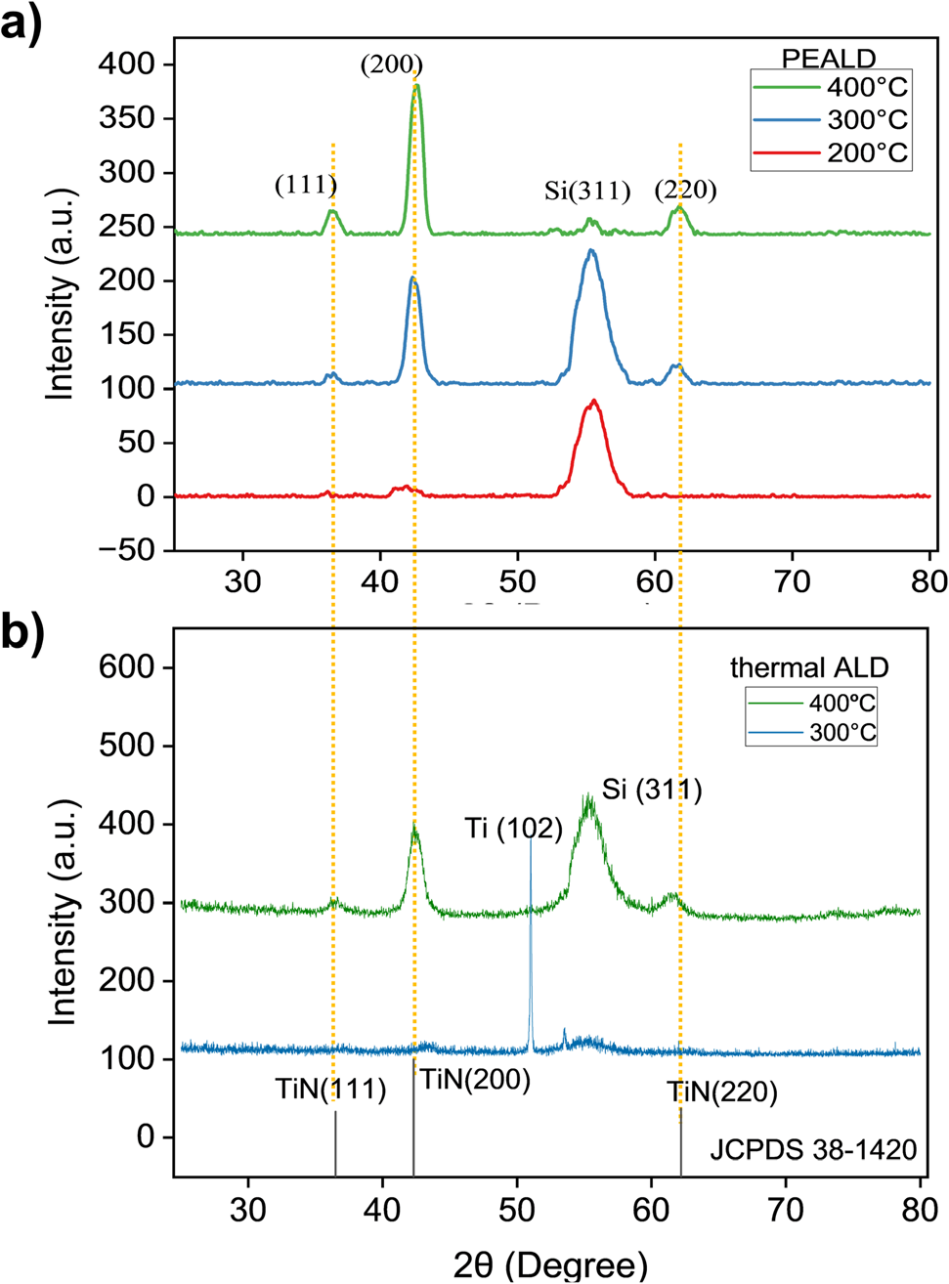
GI-XRD data of (a) PEALD TiN and (b) thermal ALD TiN grown at three different deposition temperatures.

The structural properties of the deposited film were examined by grazing incidence X-ray diffraction (GIXRD) measurements. XRD patterns in Fig. 2 of TiN thin films deposited by PEALD and thermal ALD at 200 °C, 300 °C and 400 °C revealed notable differences in crystallinity by showing phase formation and preferred orientation of the thin films depending on the method and deposition temperature. For PEALD, at 200 °C, the XRD pattern showed a broad and weak diffraction peak at (200), indicating limited crystallite formation. This might be a result of insufficient thermal energy to drive substantial atomic ordering in TiN films even if the surface reactions were chemically completed. At 300 °C, the intensity of the peaks increased, particularly for the (200) and (220) reflections at 42.6° and 61.8°, respectively, suggesting an enhancement in crystallinity through better phase formation. At 400 °C, the XRD pattern exhibited sharp and intense diffraction peaks with a dominant (200) reflection, indicative of a highly crystalline structure with a strong preferential orientation along the (200) plane. This trend suggested that increasing the deposition temperature improved the structural orientation of the TiN thin films, with 400 °C yielding the best crystallinity and a pronounced (200) orientation. The peaks corresponding to (111) at 36.7° and (220) at 61.8° confirmed the formation of cubic TiN with a rock-salt structure^42,43^ while the peak at 56.1° corresponded to the plane (311) of the Si substrate. The thermal ALD-grown film at 400 °C showed a dominant peak (200) along with two other peaks at 36.7° and 61.8°, similar to TiN grown with PEALD at the same temperature. This polycrystalline nature of PEALD and thermal ALD films was further confirmed with JCPDS 38-1420.^43^ However, the intensity of the (200) peak in the thermal ALD-grown film at 400 °C was relatively lower, indicating slightly reduced crystallinity compared to that of the PEALD-grown counterpart. At 300 °C, the XRD pattern of thermal ALD TiN films differed from that of PEALD films. Instead of showing clear diffraction peaks associated with TiN, such as the (111) and (220) reflections, the pattern exhibited a strong, high-intensity peak around 52.6°, which corresponds to metallic Ti.^44^ In addition to the dominant metallic Ti peak, the pattern showed a slight hump at around 43°, a weak indication of the presence of TiN. The absence of TiN diffraction peaks and the appearance of metallic Ti are indicative of incomplete chemical reactions at 300 °C, which are also supported by the extremely small GPC in Table 1 where at 300 °C the GPC was 0.01 Å. This means that at 300 °C the thermal ALD reactions were energetically too limited (probably due to the low reactivity of ammonia) to form TiN, and the deposited film is likely mainly composed of TiCl_4_. At 200 °C no TiN growth was observed for thermal ALD (see also Table 1), and thus no GI-XRD data possible for this thermal ALD growth temperature in Fig. 2.

Furthermore, the thermal ALD film at 400 °C showed significantly lower peak intensities for the reflections (111) and (220) compared to the Si(311) peak. This indicated a poor crystallographic orientation and a potentially higher defect density^45^ compared to the sharper peaks observed for PEALD grown films. This difference can be attributed to the plasma in PEALD, which enhances ammonia reactivity^46^ by enabling more complete and uniform surface reactions.

**Fig. 3:**
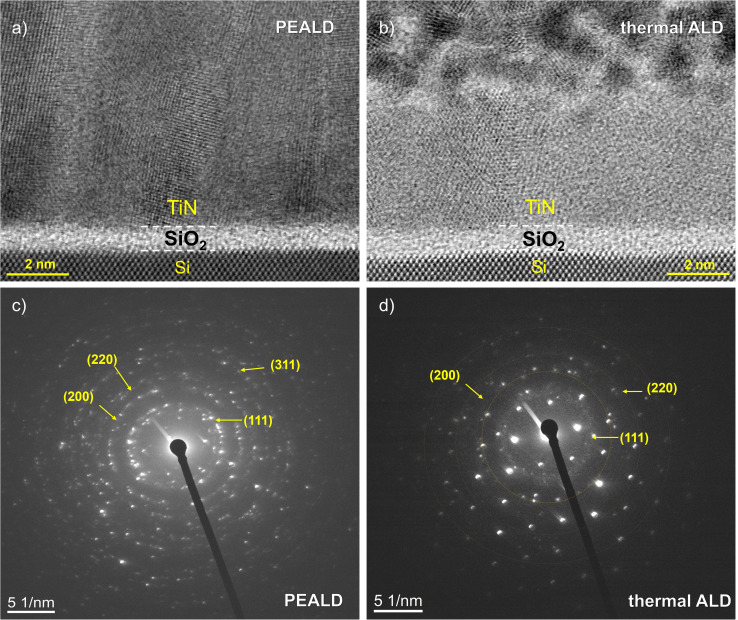
HRTEM micrographs and corresponding SAED patterns of TiN thin films deposited by PEALD and thermal ALD at 400 °C for 1200 cycles: (a) HRTEM image of the PEALD TiN film, exhibiting enhanced crystallinity and well-aligned grains; (b) HRTEM image showing TiN crystallites and atomic planes in the thermal ALD film; (c) SAED pattern of the PEALD TiN film, displaying sharp diffraction spots and well-defined rings, consistent with higher crystallinity; (d) SAED pattern of the thermal ALD TiN film, showing diffuse rings indicating lower crystallinity. The large and clear diffraction spots are superimposed diffraction from the underlying monocrystalline Si substrate, due to sample orientation in the microscope.

To further validate the XRD and ellipsometry results, high-resolution transmission electron microscopy (HRTEM) and selected area electron diffraction (SAED) analyses were performed on TiN films deposited by thermal ALD and PEALD at 400 °C for 1200 cycles. The HRTEM images shown in Fig. 3 clearly revealed microstructural differences consistent with the crystallinity trends observed in XRD. As shown in Fig. 3a, the PEALD TiN film exhibited a more crystalline and dense microstructure, characterized by distinct columnar grains that extended through a significant portion of the film thickness, consistent with previous reports.^47^ In contrast, Fig. 3b showed that the thermal ALD TiN film had a relatively disordered nanocrystalline structure in the upper regions, transitioning into a polycrystalline phase with visible grain boundaries toward the substrate interface. The grains were randomly oriented, indicating moderate crystallinity. These observations were further confirmed by the selected area electron diffraction (SAED) patterns (Fig. 3c and d). The diffraction pattern for the PEALD film (Fig. 3c) exhibited bright, sharp spots arranged in well-defined rings, indicative of a high polycrystalline structure. Distinct diffraction rings corresponding to the crystallographic planes [111], [200], [220] and [311] of TiN were clearly observed, confirming polycrystalline nature of the film and consistent with the previously reported patterns.^48^ This pattern also confirmed the high crystallinity observed in the cross-sectional TEM image, Fig. 3a. In contrast, the SAED pattern of the thermal ALD TiN film in Fig. 3d showed broader and more diffuse diffraction rings with less defined spots, reflecting a lower degree of crystallinity and greater structural disorder. This aligned with the more disordered microstructure seen in Fig. 3b. Additional sharp diffraction spots were observed that corresponded to the single crystalline silicon substrate beneath the SiO_2_ layer. These silicon spots were consistent in both samples but more dominant in the thermal ALD SAED pattern (Fig. 3d) and served as a reference. The overview of the indexed diffraction rings is presented in SI Table S1. The sharper interface and enhanced grain alignment observed in the PEALD film suggested improved film uniformity and more favorable growth kinetics, enabled by plasma assistance. HRTEM images confirming the thickness of the deposited TiN at 400 °C for 1200 cycles can be seen in SI Fig. S1. These measured thicknesses are within a few ångströms to the results of the ellipsometer recorded in Table 1, which gives great confidence in both measurements.

**Fig. 4:**
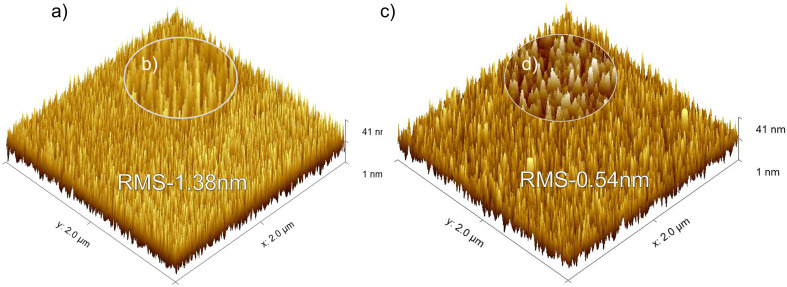
AFM topography maps of the ALD films: (a) PEALD (b) the inset highlighting a magnified section of surface illustrating the finer surface features in PEALD (c) thermal ALD (d) the inset highlighting a magnified section of the surface from thermal ALD.

To complement these structural observations, the surface morphology of the TiN films was examined using atomic force microscopy (AFM). In agreement with the HRTEM results, the PEALD TiN film exhibited a more crystalline structure. It further revealed a uniform arrangement of needle-shaped structures with a relatively consistent height distribution^49–51^ as shown in (Fig. 4a and b) for the PEALD-deposited film. In contrast, the thermal ALD deposited film as can be seen in Fig. 4c and d, showed less continuous crystal features, with gaps and variations in topography. These differences in surface morphology directly influenced the roughness of the film (RMS) presented in Table 3. PEALD films had higher roughness due to sharper and more pronounced features, while thermal ALD films exhibited lower roughness, although this roughness of thermal ALD is slightly higher than in previous studies.^44^ The lower roughness in the thermal ALD film reflected the slower and more limited reactivity in thermally driven ALD processes,^52^ which can result in incomplete crystallite growth and lower packing density, as supported by HRTEM and diffraction analyzes.

**Table 3:** RMS roughness of PEALD and thermal ALD for 1200 cycles grown at 400 °C

	PEALD	Thermal ALD
Roughness	1.38 nm	0.54 nm

### 2.2 Thermoelectric and electronic properties

The thermoelectric and electronic transport properties of TiN films grown by PEALD and thermal ALD were studied at different growth temperatures and for varying film thicknesses to understand the influence of the deposition technique on material performance.

#### 2.2.1 Effect of growth temperature

**Fig. 5:**
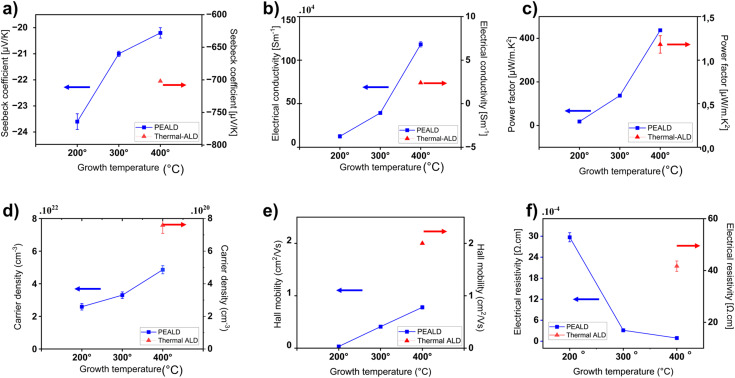
Thermoelectric and electronic properties of PEALD and thermal ALD thin films grown for 1200 cycles. (a) Seebeck coefficient; (b) electrical conductivity; (c) power factor; (d) carrier density; (e) Hall mobility and (f) electrical resistivity; the error bars reflect the 2*σ* uncertainty, derived from measurements taken at room temperature on three samples for each data point.

The analysis of PEALD TiN at different growth temperatures for 1200 ALD cycles are summarized in Fig. 5a–f and SI Table S2. A clear enhancement in all measured thermoelectric and electronic properties was observed with increasing temperature. As shown in Fig. 5a, the Seebeck coefficient became less negative with increasing temperature from −23 µV K^−1^ at 200 °C to −20.2 µV K^−1^ at 400 °C. The negative Seebeck indicates that TiN is an n-type material. This showed that there is a small reduction in the ability of the material to generate thermoelectric voltage in response to a temperature gradient. However, this reduction in the Seebeck coefficient was coupled with an increase in electrical conductivity from 3.4 × 10^4^ S m^−1^ at 200 °C to 1.1 × 10^6^ S m^−1^ at 400 °C growth temperature. This corresponds to decrease in electrical resistivity from 29.7 × 10^−4^ Ω cm to 0.91 × 10^−4^ Ω cm at respective growth temperatures. The conductivity achieved here is among the highest reported for ALD-grown TiN films compared to previous studies,^44^ demonstrating the effectiveness of PEALD. Notably, the measured resistivity is more than twice that of metallic Ti as reported in ref. 53. As a result of this synergistic effect, the power factor (PF) reached a maximum of 437 µW m^−1^ K^−2^ at 400 °C as mentioned in Fig. 5c. The power factor (PF) is a key parameter in assessing thermoelectric performance, as it indicates the amount of electrical power that can be generated per unit temperature gradient. PF is defined by the relationship (shown in eqn (1)) between the Seebeck coefficient (*S*) and the electrical conductivity *σ* in thermoelectric materials. This elevated PF of PEALD TiN highlighted the strong potential of these films for efficiently converting thermal gradients into electrical energy.

1 PF = *S*^2^*σ*

In n-type materials, the Seebeck coefficient and electrical conductivity are inversely related,^54^ primarily due to their dependence on carrier concentration. This behavior can be understood using the Mott relation,^55^ which quantitatively describes the dependence of the Seebeck coefficient on carrier concentration. According to the Mott relation:2
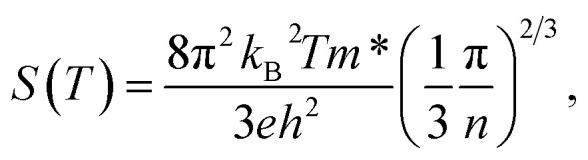
where *S*(*T*) is the Seebeck coefficient at temperature *T*, *k*_B_ is the Boltzmann constant, *e* is the electric charge, m* is the effective mass, *h* is Planck's constant and *n* is the charge carrier density. As presented in Fig. 5d, the carrier concentration increased from 2.59 × 10^22^ cm^−3^ at 200 °C to 4.86 × 10^22^ cm^−3^ at 400 °C. This increase in carrier density resulted in a decrease of the measured Seebeck coefficient at higher deposition temperatures, which followed the trend predicted by the Mott relation. This was because a higher carrier concentration reduces the thermoelectric voltage generated across the material when subjected to a temperature gradient.^42,56^

The PEALD films also showed a significantly higher carrier concentration than the thermal films. This is attributed to a high density of intrinsic nitrogen vacancies which act as double donors. The energetic plasma environment efficiently dissociates precursor molecules, generating highly reactive species (radicals, ions) which provide substantial energy at the growing surface and remove ligands, creating a nitrogen deficiency. These contribute to much higher electrical conductivity in PEALD films compared to thermal ALD, as shown in Fig. 5b. As revealed by AFM analysis, PEALD films had a surface roughness higher than that of thermal ALD films, causing more surface scattering which, when coupled with a higher carrier concentration, resulted in increased carrier–carrier and surface-related scattering. As a result, despite their high conductivity, PEALD films exhibited reduced Hall mobility compared to thermal films, as seen in Fig. 5e. However, with increasing growth temperature, the Hall mobility of PEALD films improves substantially from 0.032 cm^2^ V^−1^ s^−1^ at 200 °C to 0.78 cm^2^ V^−1^ s^−1^ at 400 °C as shown in (Fig. 5e). This improvement was likely due to better crystallinity and reduced defect scattering due to higher growth temperatures. In contrast, thermal ALD films showed no or minimal growth at 200 °C and 300 °C, limiting their electrical characterization at these lower growth temperatures as the films did not grow or were too thin to be characterized. At 400 °C, however, thermal ALD films exhibited a smoother surface morphology, as confirmed by AFM, which effectively reduces surface scattering. Furthermore, the lower carrier concentration in these films (Fig. 5d) might further limits carrier–carrier interactions. These factors contribute to a longer mean free path for charge carriers and improved Hall mobility reaching 2.0 cm^2^ V^−1^ s^−1^ at 400 °C as shown in Fig. 5e, despite the lower overall conductivity compared to PEALD films.^42^

In terms of thermoelectric performance, the Seebeck coefficient of PEALD films at 400 °C was relatively low, measured at −20 µV K^−1^, whereas thermal ALD films exhibited a significantly higher value of −702 µV K^−1^. These results were aligned with the Mott relation in eqn (2). The corresponding carrier concentration trends shown in Fig. 5d, where thermal ALD films possess a carrier concentration much lower than that of PEALD films. The lower carrier concentration in thermal ALD films enhanced the energy-dependent asymmetry in carrier distribution, resulting in a significantly higher Seebeck coefficient in contrast to PEALD. This highlighted a stronger thermoelectric potential in thermal ALD films grown at 400 °C, as a more negative Seebeck coefficient is a characteristic of improved thermoelectric behavior for n-type materials. However, despite this advantage, the electrical conductivity for thermal ALD films at 400 °C remained considerably lower than that of PEALD films at the same temperature. As a result when grown at 400 °C, the overall power factor of thermal ALD films was lower, measured at 1.18 µW m^−1^ K^−2^, in contrast to the much higher power factor of 437 µW m^−1^ K^−2^ observed for PEALD films. This reflects the trade-off between Seebeck coefficient and electrical conductivity in determining thermoelectric performance. A complete set of electrical and thermoelectric data supporting these observations is provided in SI Table S3.

#### 2.2.2 Effect of number of cycles

**Fig. 6:**
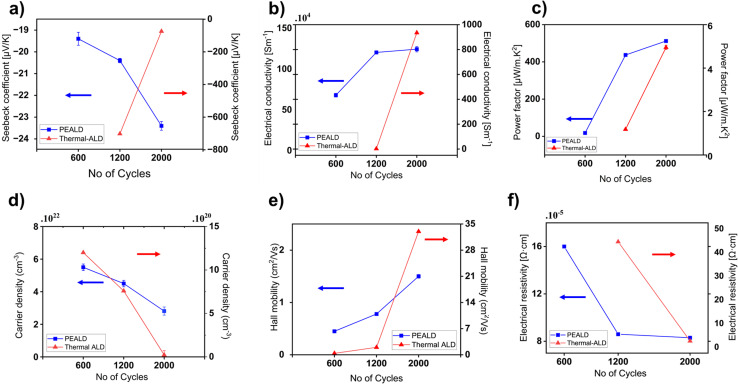
Thermoelectric and electronic properties of PEALD and thermal ALD thin films with varied number of ALD cycles grown at 400 °C. (a) Seebeck coefficient; (b) electrical conductivity; (c) power factor; (d) carrier density; (e) Hall mobility and (f) electrical resistivity. The error bars reflect the 2*σ* uncertainty, derived from measurements taken at room temperature on three samples for each data point.

**Fig. 7:**
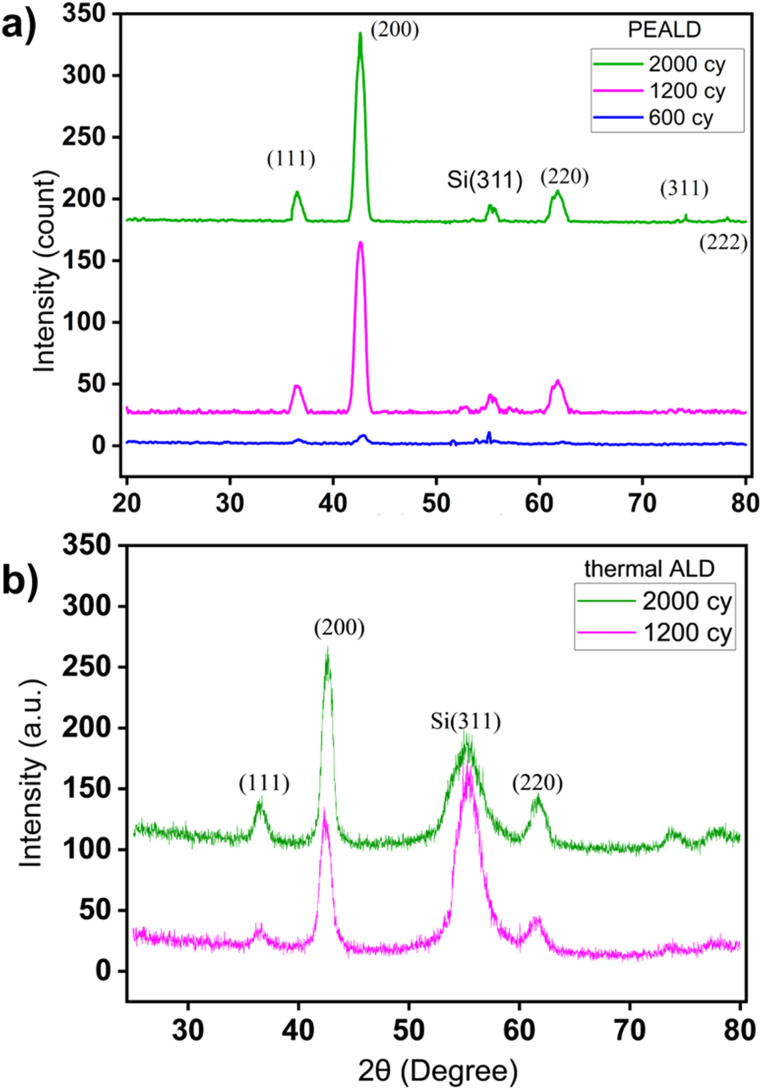
GI-XRD image of (a) PEALD and (b) thermal ALD TiN grown for different number of ALD cycles.

As the number of ALD cycles increased from 600 to 2000, the carrier concentration in PEALD TiN films decreased from 5.74 × 10^22^ cm^−3^ to 2.8 × 10^22^ cm^−3^, as shown in Fig. 6d. This trend is attributed to the progressive development of phase formation and structural ordering, as evidenced by the increasing intensity of TiN diffraction peaks of (111), (200), and (220) in the GIXRD data (Fig. 7a). In many transition metal nitrides, including TiN, structural defects such as nitrogen vacancies and dislocations can act as unintentional n-type dopants.^57^ Therefore, improved structural ordering with higher cycle numbers reduces defect density, leading to lower intrinsic carrier concentrations. According to the Mott relation, a decrease in carrier concentration leads to an increase in the Seebeck coefficient absolute value. This is confirmed by the observed increase in absolute Seebeck from −19 µV K^−1^ to −23 µV K^−1^(as shown in Fig. 6)a, suggesting an enhancement in the thermoelectric voltage generation capability. The higher absolute value of the Seebeck coefficient reflected an improved energy filtering effect and stronger n-type behavior, where fewer but more energetically selective carriers contribute to the thermoelectric response.

At the same time, as the carrier density decreased with increase number of cycles, the Hall mobility for PEALD TiN increased from 0.5 cm^2^ V^−1^ s^−1^ to 1.5 cm^2^ V^−1^ s^−1^ as shown in Fig. 6e. This can be attributed to reduced defect scattering due to better phase formation and structural orientation in TiN films, as shown in Fig. 7a, with increased number of cycles. Despite the drop in carrier concentration, the electrical conductivity of PEALD TiN almost doubled from 600 to 2000 cycles (Fig. 6b). This strongly supports the notion that with the improved structural orientation, higher mobility compensates for the lower carrier density, resulting in an overall enhanced conductivity. Finally, the power factor increased from 291 µW m^−1^ K^−2^ to 512 µW m^−1^ K^−2^, driven by eqn (1), leading to better thermoelectric performance in the PEALD TiN films.

A similar trend in phase evolution of thermal TiN thin films grown at 400 °C is shown in Fig. 7b, with increasing ALD cycles from 1200 to 2000. The 600-cycle film (5.8 nm) was below the XRD detection limit. At 1200 cycles, weak peaks corresponding to the (111) and (220) orientations were visible. This suggested that the films were still not yet fully crystallized and likely to contain more structural defects. This contributed to the relatively high Seebeck coefficient of −702 µV K^−1^, as shown in Fig. 6a. With improved phase formation at 2000 cycles, defect density decreases, resulting in reduced scattering and a lower Seebeck value of −72 µV K^−1^.

Furthermore, improvement in structural quality of the thermal ALD TiN at higher cycle numbers suppress scattering from defects, surfaces, and between carriers themselves. This leads to an increase in the mean free path of charge carriers and enhanced Hall mobility from 2 cm^2^ V^−1^ s^−1^ to 33 cm^2^ V^−1^ s^−1^ as seen in Fig. 6e. This showed that charge carriers move more freely through the film. As a result, electrical conductivities improved, increasing from 2.4 S m^−1^ at 1200 cycles to 1000 S m^−1^ at 2000 cycles as seen in Fig. 6b. This together improved the power factor shown in eqn (1), from 1.2 µW m^−1^ K^−2^ to 4.95 µW m^−1^ K^−2^ at 2000 cycles (Fig. 6c). Although this indicated more efficient charge transport because of better film quality, it also illustrated a well-known trade-off in thermoelectric properties: as crystallinity and carrier mobility improved, the magnitude of the Seebeck coefficient tended to decrease. This is because fewer energetic asymmetries remain to generate the thermoelectric voltage, even when the carrier concentration remains low. Therefore, careful optimization is required to balance Seebeck coefficient, conductivity, and mobility in order to maximize overall thermoelectric performance.

### 2.3 Thermal properties and thermoelectric figure of merit

The thermal conductivity (*κ*) of the TiN films deposited by both PEALD and thermal ALD was measured using a thin film laser flash analyzer (TF-LFA), which required a minimum film thickness of approximately 80 nm. Consequently, these measurements were performed on thicker films fabricated using a greater number of ALD cycles. The measured thermal conductivity value at room temperature for 86 nm thin films was 26.96 W m^−1^ K^−1^ for PEALD TiN and 7.01 W m^−1^ K^−1^ for thermal ALD TiN. In the fitting model used for the data analysis, the bulk values of the density (*ρ* = 5450 kg m^−3^) and the specific heat capacity (*C*_p_ = 636 J kg^−1^ K^−1^) of TiN^58^ were implemented. Details of the multilayer heat transfer model and the fitting curves are provided in SI. To the best of our knowledge, these are the first reported values of thermal conductivity for ALD-deposited TiN thin films. Although the exact thermal conductivity of the thinner TiN films used in the thermoelectric characterization could not be measured directly, lower values are reasonable due to the increased phonon scattering at film boundaries and interfaces, which becomes more pronounced at reduced thicknesses.^59,60^ This scattering disrupts the coherent propagation of phonons, reducing their ability to transfer heat effectively. Therefore, the thermal conductivity values obtained from the thicker films can be used as an estimate of the maximum (*κ*) value possible for thinner TiN films. With both the thermoelectric power factor and the thermal conductivity measured at room temperature, we can now calculate the thermoelectric figure of merit (*zT*) using relation shown in eqn (3):3
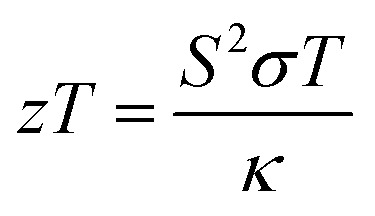


For PEALD TiN, we obtained a room-temperature *zT* value of 0.0056 for films deposited using 2000 ALD cycles at 400 °C, while the corresponding value for thermal ALD TiN under the same deposition conditions was 0.0002 showing that PEALD significantly enhances the thermoelectric performance of TiN films due to improved electrical conductivity.

## 3 Experimental

### 3.1 Sample preparation

Thin films of ALD TiN were deposited on two types of substrates: Si and soda-lime glass. The Si substrates had dimensions of 10 mm × 10 mm, while two sizes of soda-lime glass substrate samples were prepared: 4 mm × 20 mm and 10 mm × 10 mm. Prior to deposition, all substrates were ultrasonically cleaned in acetone, isopropyl alcohol (IPA), and deionized water (DIW) for 5 minutes at room temperature, then dried under N_2_ flow to ensure surface cleanliness. The depositions were carried out in a Picosun R200 Advanced system using two different ALD processes: plasma-enhanced ALD (PEALD) and thermal ALD. Titanium tetrachloride TiCl_4_ (Volatec, >99.5%) and ammonia NH_3_ (Linde, >99.5%) were used as precursors with N_2_ as carrier gas and purge gas. The N_2_ flow was set to 80 sccm in the TiCl_4_ line for both PEALD and thermal ALD while the NH_3_ line flow was 90 sccm for PEALD and 60 sccm for thermal ALD. The deposition parameters for the ALD TiN films are summarized in Tables 4 and 5. The chamber pressure during the deposition was maintained below 1 mbar. In the PEALD process, an inductively coupled plasma (ICP) with the power of 2500 W was applied during the NH_3_ exposure step. Specifically, the plasma was ignited for 3 s along within a pulse of 4.5 s NH_3_ to activate NH_3_ in reactive nitrogen species to efficiently remove chlorine ligands from the surface and facilitate the formation of the Ti–N bond. In both methods, the deposition temperatures ranged from 200 °C to 400 °C to study their impact on the thermoelectric performance of the films. Based on the process parameters of ALD-TiN followed in a previous study,^61^ TiN films with targeted thicknesses of 15 nm, 30 nm and 50 nm were deposited at 400 °C. The number of ALD cycles used to target each film thickness for both the PEALD and thermal processes is detailed in Table 6. After deposition, the samples were kept in the load lock for 20 minutes to cool before being removed to prevent oxidation.

**Table 4:** The process parameters for depositing ALD TiN films

Process parameter	Specification
Precursor 1	TiCl_4_
Precursor 2	NH_3_
Carrier gas	N_2_
Pressure	<1 mbar
Temperature	200–400 °C
Substrate	Glass and Si chips

**Table 5:** ALD process sequence for thermal and PEALD techniques

ALD process	TiCl_4_	NH_3_
	Pulse/purge/flow	Pulse/purge/flow
PEALD	0.1 s/2 s/90 sccm	4.5 s/6 s/80 sccm
Thermal ALD	0.1 s/2 s/60 sccm	0.1 s/2 s/80 sccm

**Table 6:** Number of ALD cycles and corresponding targeted thickness for PEALD and thermal ALD of TiN films, referenced from previous study^61^

ALD process	Number of cycles		
	15 (nm)	30 (nm)	50 (nm)
PEALD	600	1200	2000
Thermal ALD	1200	2000	4000

### 3.2 Structural characterization

The surface characterization of ALD TiN films was performed using Si substrate samples. The film thickness was measured using a spectroscopic ellipsometer SE-2000 (Semilab). The measurement was done in the mapping mode by averaging five positions on the sample surface. The surface characterization of the sample was further performed by using atomic force microscopy (AFM-Bruker Dimension Icon). The XRD patterns of the films were obtained using Grazing Incidence X-ray Diffraction (GIXRD) on a Rigaku SmartLab diffractometer with copper Kα radiation (*λ* = 1.5418 Å), covering a 2*θ* range of 20° to 80°. Furthermore, thin cross-section lamellae were cut from TiN films deposited on the silicon substrate with focused ion beam microscopy (FIB-SEM) using JEOL JIB-4700F. High resolution transmission electron microscopy (HR-TEM) was performed using lamellae prepared from the films, with a JEOL JEM-2800 microscope operated at an accelerating voltage of 150 kV. The microstructure was studied through HR-TEM imaging and selected area electron diffraction (SAED).

### 3.3 Electrical and thermoelectric characterization

The in-plane resistivity and the Seebeck coefficient were measured using a thin film adapter on an LSR-3 (Linseis) system in He atmosphere with five temperature gradients with a stabilization time of 10 min for each gradient. Thermoelectric analysis was performed on TiN films grown on 4 × 20 mm^2^ glass substrates. The LSR-3 measurement details and the comprehensive analysis of the film properties are thoroughly discussed in a previous study.^62^ Finally, the Hall mobility (*µ*) and carrier density of the samples were determined using electrical contacts on a 10 mm × 10 mm glass substrate samples, with measurements performed in the van der Pauw configuration on a Hall Measurement System (HMS-5300, Ecopia). All measurements were performed at room temperature.

### 3.4 Thermal characterization

The thermal conductivity of TiN thin films was measured using a nanosecond transient thermoreflectance (TTR) technique *via* a thin film laser flash analyzer (TF-LFA, Linseis). This optical pump–probe system utilizes a Q-switched pulsed Nd:YAG laser of 1064 nm for thermal excitation, and a 476 nm diode laser was used as the probe to monitor the resulting temperature transients. Before measurement, a Ti/Au transducer layer (20 nm/200 nm respectively) was deposited onto the 10 mm × 10 mm sample surface *via* electron beam evaporation. The thickness of the transducer layer was measured using a stylus profilometer (Bruker Dektak XT) and confirmed to 17/176 nm to ensure accurate thermal modeling. TTR signals as a function of time were recorded from the surface of the Au layer and averaged over 80 pump laser pulses at five different spatial locations per sample at room temperature to ensure reliable spatial averaging. Data fitting was performed using a multilayer heat transfer model to extract the in-plane thermal conductivity of the TiN film.

## 4 Conclusions

This study conducted a detailed analysis of the thermoelectric, electronic transport, and thermal properties of TiN films grown by PEALD and thermal ALD, highlighting distinct trends influenced by growth temperature, film thickness, and process type. TiN films grown by PEALD at 400 °C for 2000 cycles (thickness-50.6 nm) exhibited superior thermoelectric performance at room temperature compared to thermal ALD films. PEALD TiN films achieved a higher electrical conductivity of 1.17 × 10^6^ S m^−1^ and a power factor of 512 µW m^−1^ K^−2^, despite a moderate Seebeck coefficient of −23 µV K^−1^ due to increased carrier concentration of 2.81 × 10^22^ cm^−3^. The films benefited from the enhanced number of charge carriers but suffered from a reduced Hall mobility of 1.5 cm^2^ V^−1^ s^−1^. The thermal conductivity of the thicker PEALD TiN film (86 nm) was measured as 26.95 W m^−1^ K^−1^ and based on this value, the *zT* for the PEALD film of 50.6 nm was calculated at 0.0056. In contrast, thermal ALD films grown at the same temperature showed a higher Seebeck coefficient −73 µV K^−1^, attributed to a lower carrier concentration of 1.38 × 10^19^ cm^−3^. However, this resulted in a lower electrical conductivity 1000 S m^−1^ with a power factor of 4.95 µW m^−1^ K^−2^. With a measured thermal conductivity of 7.01 W m^−1^ K^−1^, the corresponding *zT* value for the thermal ALD film measured as 0.0002.

This study provides the first in-depth investigation of thermoelectric and electronic properties of ALD TiN films, addressing a previously unexplored area. The results are crucial in optimizing these properties in the design and development of ALD TiN-based thin films for thermoelectric and energy conversion applications. Furthermore, the data presented here enable ALD TiN to be implemented in various nanotechnology applications as crucial material parameters are now known.

## Author contributions

Conceptualization, C. K. and P. G.; methodology, P. G. and C. K.; investigation, P. G. and R. R.; analysis, P. G. and C. K.; supervision, C. K. and I. T.; writing – original draft, P. G.; writing – review and editing, R. R., C. K. and I. T.

## Conflicts of interest

There are no conflicts to declare.

## Supplementary Material

NA-OLF-D5NA00914F-s001

## Data Availability

All data supporting the findings of this study are provided in the supplementary information (SI) file associated with this article. No additional datasets were generated or analyzed beyond those included in the SI. Supplementary information: all the observed data is tabulated. See DOI: https://doi.org/10.1039/d5na00914f.
