# Supplementary material for: Thermoelectric and electronic transport properties of thermal and plasma-enhanced ALD grown titanium nitride thin films
Source: Nanoscale Adv. 2025 Dec 4;8(2):612–22. doi: 10.1039/d5na00914f (PMC12687283; doi:10.1039/d5na00914f)
Supplement: NA-008-D5NA00914F-s001 [file NA-008-D5NA00914F-s001.pdf]

Supplementary Information (SI) for Nanoscale Advances.
This journal is © The Royal Society of Chemistry 2025

SUPPLEMENTARY INFORMATION

Thermoelectric and Electronic Transport Properties of Thermal and Plasma-Enhanced ALD Grown Titanium Nitride Thin Films

P.Goel*[a], C. Kauppinen[b], R. Raju[a], and I. Tittonen[a]

a. Department of Electronics and Nanoengineering, Aalto University, Espoo, 00076 AALTO, Finland

b. VTT Technical Research Centre of Finland Ltd., Espoo, FI-02044 VTT, Finland

*E-mail: priyanka.goel@aalto.fi

Table S1: Selected Area electron diffraction measurements. The radius was measured using ImageJ software.

TiN crystal orientations	Radius (nm)-PEALD	Radius (nm)-Thermal ALD
**[111]**	4.25±0.2	4.77±0.03
**[200]**	5.01±0.01	5.15±0.1
**[220]**	7.110±0.1	7.52±0.4
**[311]**	10.84±0.1	11.34±0.1

d-spacing for the diffraction planes were measured from radius using formula, d = 1/radius. This d-spacing is then used for indexing rings.

Table S2: Variation of the thermoelectric properties of PEALD TiN thin films grown at different temperature

Growth temp (C)	Thickness TiN(nm)	Seebeck coefficient (µV/K)	Conductivity x 10^4 (Sm^{-1})	PF (µW/mK ^{2})	Hall mobility (cm^2/Vs)	Carrier density (cm^{-3})	Resistivity x 10^{-4} (Ω cm)
**200°**	20±3	-23.6±0.003	3.37±2.3	18.1±0.8	0.032±0.002	2.59 x10 ^{22}	29.7
**300°**	31±2,7	-21.0±0.001	32±0.1	137±3	0.41±0.02	3.3 x10 ^{22}	3.13
**400°**	31±2	-20.2±0.002	116±3.3	437±4	0.78±0.04	4.86 x10 ^{22}	0.91

Table S3: Variation of the thermoelectric properties of thermal ALD TiN thin films grown at different temperature

Growth temp (C)	Thickness TiN(nm)	Seebeck coefficient (µV/K)	Conductivity x 10^4 (Sm^{-1})	PF (µW/mK ^{2})	Hall mobility (cm^2/Vs)	Carrier density (cm^{-3})	Resistivity (Ω cm)
**200°**	**No growth**	-	-	-	-	-	-
**300°**	1.2 ±0.8	-	-	-	-	-	-
**400°**	12.0±2	-702±0.007	2.4±0.02	1.18±0.1	2±0.02	7.59 x10 ^{20}	41.7±2

Table S4: Variation of the thermoelectric properties of PEALD TiN thin films grown at 400°C with number of ALD cycles

No. of cycles	Thickness TiN(nm)	Seebeck coefficient (µV/K)	Conductivity x 10^4 (Sm^{-1})	PF (µW/mK ^{2})	Hall mobility (cm^2/Vs)	Carrier density (cm^{-3})	Resistivity x 10^{-5}(Ω cm)
**600**	17.7±3	-19.4±0.3	63.6±2.3	18.4±0.8	0.45±0.002	5.59 x10^{22}	16
**1200**	31±2	-20.2±0.002	116±3.3	437±4	0.78±0.04	4.86 x10^{22}	8.6
**2000**	50.4±1.5	-23.4±0.2	120±2	512±4	1.5±0.04	2.81 x10^{22}	8.3

Table S5: Variation of the thermoelectric properties of thermal ALD TiN thin films grown at 400°C with number of ALD cycles

No. of cycles	Thickness TiN(nm)	Seebeck coefficient (µV/K)	Conductivity (Sm^{-1})	PF (µW/mK ^{2})	Hall mobility (cm^2/Vs)	Carrier density (cm^{-3})	Resistivity (Ω cm)
**600**	5.8	-	-	-	0.4±0.002	12 x10^{20}	-
**1200**	12±2	-702±0.007	2.4±0.02	1.18±0.1	2±0.02	7.59 x10^{20}	42±1
**2000**	24.1±0.7	-74.3.4±0.7	935±3.3	4.95±0.2	33±0.02	0.22 x10^{20}	0.11±0.02

Figure S1: HRTEM images confirming the thickness of thermal ALD and PEALD TiN grown with 1200 cycles at 400°C

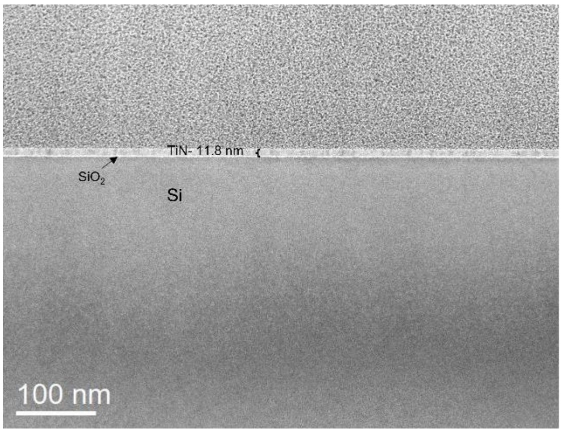

a) Thermal-ALD

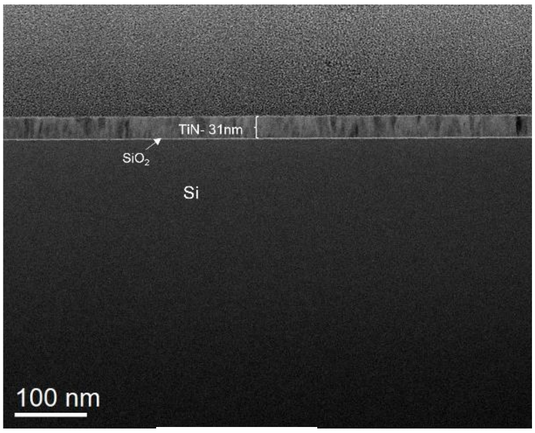

b) PEALD

Table S6: Other details of the PEALD TiN sample used for fitting the model:

Material	Thickness (nm)	Cp (J/kgK)	rho (kg/dm^3)	K (W/mK)
Au	176,00	129,00	19,30	317,00
Ti	17,00	522,00	4,51	21,90
Si	500000,00	703,00	2,34	150,00

Density (TiN): 5,40 kg/dm^3

Specific heat capacity for TiN: 636,00 J/kgK

For PEALD TiN Film-

Thermal Conductivity (W/m·K) after fitting: 26.96
Film Thickness (nm): 86.00
R^2 Value: 0.953935

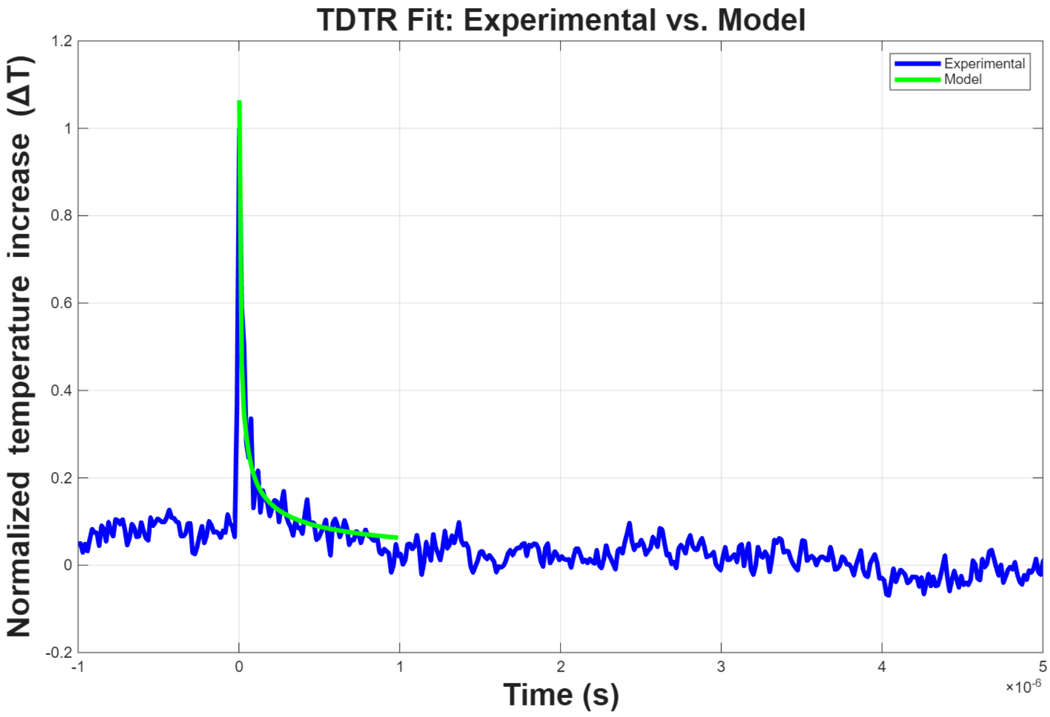

Figure S2: Thermal Conductivity decay curve for PEALD TiN after fitting

For thermal ALD TiN Film-

Thermal Conductivity (W/m·K) after fitting: 7.01

Film Thickness (nm): 86.5

R^2 Value: 0.97

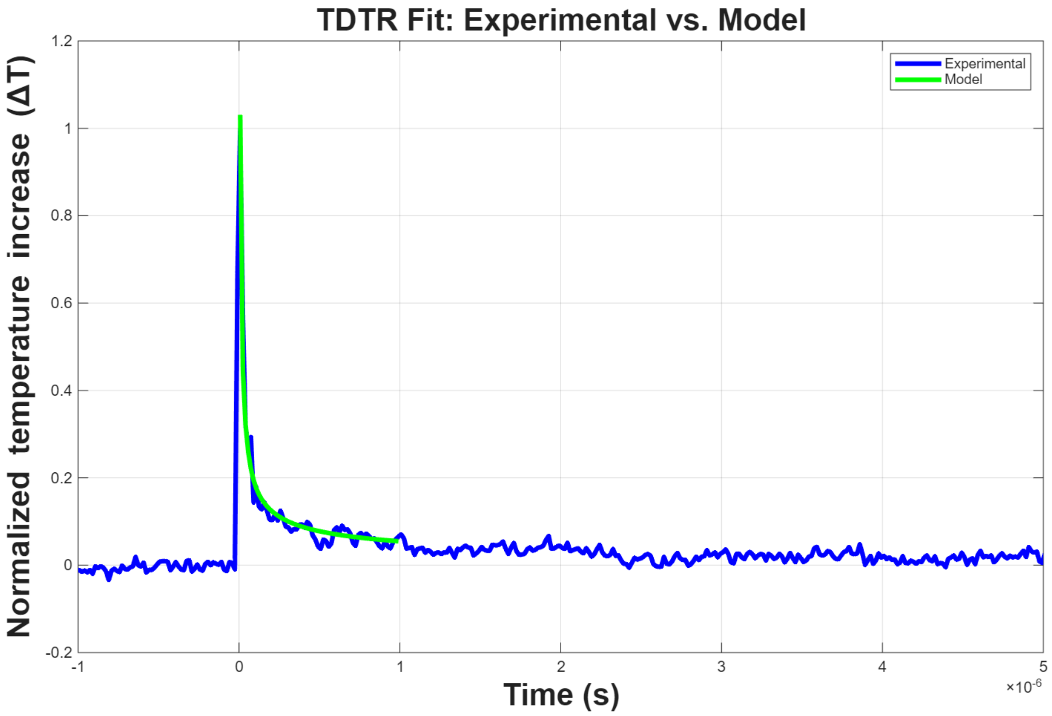

Figure S3: Thermal Conductivity decay curve for thermal ALD TiN
